# Localized fluid discharge by tensile cracking during the post-seismic period in subduction zones

**DOI:** 10.1038/s41598-020-68418-z

**Published:** 2020-08-03

**Authors:** Makoto Otsubo, Jeanne L. Hardebeck, Ayumu Miyakawa, Asuka Yamaguchi, Gaku Kimura

**Affiliations:** 1 Geological Survey of Japan, AIST, Tsukuba, 305-8567 Japan; 2 U.S. Geological Survey, Menlo Park, 94025 USA; 3 Atmosphere and Ocean Research Institute, The University of Tokyo, Kashiwa, 277-8564 Japan; 4 Tokyo University of Marine Science and Technology, Tokyo, 108-8477 Japan

**Keywords:** Hydrology, Solid Earth sciences

## Abstract

It is thought that extensional structures (extensional cracks and normal faults) generated during the post-seismic period create fluid pathways that enhance the drainage of the subducting plate interface, thus reducing the pore pressure and increasing fault strength. However, it remains to be elucidated how much pore fluid pressure decreases by the extension crack formation. Here we examined (i) the pore fluid pressure decrease, and (ii) the degree fault strength recovery by the extension crack formation during the post-seismic period by analyzing extension quartz veins exposed around the Nobeoka Thrust, southwestern Japan. The Nobeoka Trust is an on-land analog of the modern splay fault at shallow depths (~ 8 km) in the Nankai Trough. The poro-elastic model of extensional quartz vein formation indicates that the formation of extensional cracks only releases up to ~ 7–8% of the total pore fluid pressure at ~ 8 km depth. The pore pressure around the Nobeoka Thrust was close to lithostatic pressure during the entire seismic cycle. The estimated effective frictional coefficient along the Nobeoka Thrust after this small fluid-loss by the extensional crack formation does not exceed 0.15. Hence, the pore fluid pressure reduction due to the post-seismic extensional cracks contributes little to increase the fault strength of the megasplay fault.

## Introduction

Pore fluid pressure (*P*_f_) is of great importance in understanding earthquake mechanics. The temporal buildup of pore fluid pressure during the seismic cycle may promote temporal changes in fault strength^1^. In the co-seismic period of the seismic cycle, high pore fluid pressure close to lithostatic is observed around faults (i.e., where the pore fluid pressure ratio, *λ*_v_ = *P*_f_/σ_v_ > 0.9; σ_v_ vertical stress^2,3^). The distribution of high pore fluid pressure is likely to be time-dependent, thus varying over the seismic cycle^1^. The time-varying seismic reflectivity may be used to detect the porosity changes related to the development of pore fluid overpressure along the subduction interfaces^4^. The porosity changes are controlled by the temporal stress changes during the seismic cycle. In forearc regions, the near complete stress release that occurs during huge trench-type earthquakes (e.g., the 2011 Tohoku-oki earthquake) induces a change from reverse-faulting type stress regime to a non-Andersonian stress regime including normal faulting earthquakes^5^. Previous hydrological research has suggested that the fluid loss by the formation of these extensional deformation structures (e.g., extension cracks and normal faults) in the post-seismic period increases the fault strength and creates drainage asperities along the plate interface^6^. Here, we focus on the fluid migration in the hanging wall by the extension crack formation. The key question arises: To what degree is pore fluid pressure reduced by the extension crack formation? The temporal change in pore fluid pressure given by Skempton's relationship^7,8^ is of the same order as the stress drop related to trench type earthquakes^6^. However, crustal stresses and pore fluid pressures at depth are difficult to quantify directly, and downhole measurements of in situ pore fluid pressure are generally limited to depths of a few kilometers^9^. Sibson^6^ estimated that the change in frictional strength at 10 km depth is 6–64 MPa, increasing to 26–256 MPa at 40 km depth. Given the large range of estimates at each depth range, we seek to more tightly constrain the change in pore fluid pressure and the resulting change in frictional strength.

To answer this question, we examine temporal changes in pore fluid pressure during seismic cycles by analyzing extension mineral veins that are exposed along an ancient megasplay fault. The Nobeoka Thrust, southwestern Japan, which is an on-land example of the megasplay fault at shallow depths in the Nankai Trough, contains datasets of quartz veins that enable an understanding of fluid pathways in the hanging wall of the subduction zone^10–13^. Mineral veins are the fossils of the ancient fluid flow, as widespread quartz veins in subduction zones at seismogenic depths are generally taken as evidence of significant fluid flow and silica precipitation^14–16^. The silica precipitation in the accretionary wedge reduces the rock porosity and may control the recurrence interval of large earthquakes in subduction zones^17,18^. In this study, by using the poro-elastic model for extension quartz vein formation^19^, we estimate (i) the pore fluid pressure loss and (ii) the amount of fault strength recovery by the extension crack formation during the post-seismic period in a subduction zone.

## Model for estimating pore fluid overpressure

The character of the tectonic stress regime (i.e., the principal compressive stresses, σ_1_ > σ_2_ > σ_3_) plays a critical role in the containment of pore fluid overpressure, with overpressures being much more easily sustained in compressional stress fields^20^. Brittle-rock failure depends on the coefficient of internal friction and the balance between differential stress (Δσ = σ_1_ − σ_3_) and rock tensile strength (*Ts*)^21–23^. In particular mode I cracks, which we focused on this study, is formed under low differential stress of Δσ < 4*Ts*^21^. At the same time, the type of failure affecting an intact rock mass is strongly dependent on *P*_f_, which controls the principal effective stresses^24^. For fluid to generate a crack, *P*_f_ must exceed the normal stress (σ_n_) acting on the crack wall^25^. Extensional veins are formed when pore fluid pressure exceeds the sum of the minimum principal stress (σ_3_) and *Ts*^26^. Hence, the extensional veins are formed when

1 $$ P_{{\text{f}}} > \sigma_{{3}} + Ts. $$

**Figure 1:**
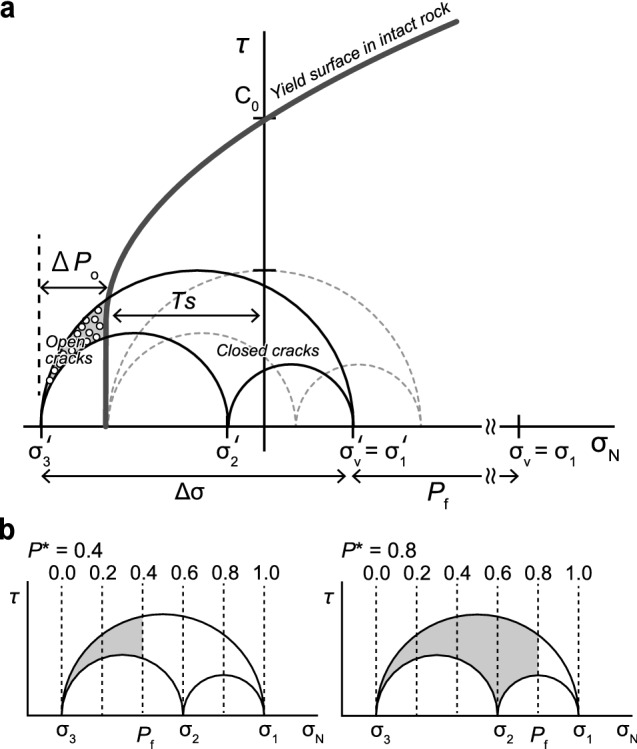
Mohr diagram. (**a**) Schematic illustrations showing formation of extension cracks by using the Mohr diagram. Open circles inside the shaded area indicate the normal and shear stress magnitudes on the cracks. When the pore fluid pressure exceeds the sum of σ_3_ and the tensile strength (*Ts*), veins filling mode I cracks are generated. When high-pressurized fluids flow into the mode I cracks, the overpressure is reduced (Gray dotted Mohr diagram). *P*_f_: pore fluid pressure. Δ*P*_o_: pore fluid overpressure. Δσ: differential stress for formation of mode I cracks. σ**′**: effective stress. σ_N_: normal stress. *τ*: shear stress. σ_v_: over burden pressure. C_0_: cohesion. *Ts*: rock tensile strength. (**b**) Pore fluid pressure assumed to satisfy P* = (*P*_f_ − σ_3_ − *Ts*)/(σ_1_ − σ_3_) = 0.4 (left) and 0.8 (right). Gray regions show the pore fluid pressure exceeds σ_3_. σ_N_: normal stress. τ: shear stress.

The mechanics of crack opening can be represented in three dimensions using the Mohr circle construction, with the conditions for opening being represented by the shaded area in Fig. 1. The part of *P*_f_ that exceed the σ_3_ + *Ts* sum is the pore fluid overpressure (Δ*P*_o_) defined as

2 $$\Delta P_{{\text{o}}} = \left( {P_{{\text{f}}} - \left( {\sigma_{{3}} + Ts} \right)} \right) = P_{{\text{f}}} - \sigma_{{3}} - Ts.$$

Here, we employed the poro-elastic model to estimate Δ*P*_o_. To calculate the Δ*P*_o_ is possible to use the vein aspect ratio (*W*/*L*), where *W* represent vein aperture width and *L* the vein length, assuming that it is linearly related to pore fluid overpressure Δ*P*_o_ and to the elastic rock properties of the host rock^19^:

3 $$ W/L = \left( {\Delta P_{{\text{o}}} {2}\left( {{1} - \nu^{{2}} } \right)} \right)/E $$

where *ν* is the Poisson’s ratio and *E* the Young’s modulus. Hence, *W*/*L* of veins is depended on Δ*P*_o_ and *E*^19^.

The driving pore fluid pressure ratio (P*) is the ration between the pore fluid overpressure Δ*P*_o_ and Δσ as follows:

4 $$P^* = \Delta P_{{\text{o}}} /\Delta \sigma = (P_{{\text{f}}} - \sigma_{{3}} - Ts)/\left( {\sigma_{{1}} - \sigma_{{3}} } \right).$$

P* is estimated as the result of the stress tensor inversion using the veins^27,28^. In nature, there is both type of the vein: the vein filling re-opened crack and the vein filling newly formed crack. It is easily re-opened the existing cracks (*Ts* = 0) under lower pore pressure than the pore pressure to newly form the cracks. Therefore, to estimate the upper limit of the pore pressure, considering the formation of the crack is appropriate and we use the equation with *Ts* on P*. P* varies from 0 (no opening of cracks) to 1 (forming new cracks), and describes the equilibrium between *P*_f_ and the minimum and maximum stresses (Fig. 1). P* is estimated by picking the maximum normal stress among all veins in a Mohr circle^28,29^. P* means the mode I cracks are formed as a result of multiple ascending events of fluids with various fluid pressures^28^. Here, we assume that Δ*P*_o_ is reduced when the high-pressure fluid flows into the mode I cracks.

We can thus calculate Δσ from the driving pore fluid pressure ratio P* and the pore fluid overpressure Δ*P*_o_ rearranging Eq. (4):

5 $$\Delta\sigma = \sigma_{{1}} - \sigma_{{3}} = {\Delta}P_{{\text{o}}} /P^*.$$

Thus, we can thus calculate *P*_f_ arranging Eq. (2) as follows:

6 $$P_{{\text{f}}} = \sigma_{{3}} + Ts + {\Delta}P_{{\text{o}}} .$$

In particular, Δσ = σ_1_ − σ_3_, therefore σ_3_ = σ_1_ − Δσ. If the extensional veins are formed under a normal faulting type stress regime, σ_1_ = σ_v_ calculated as *ρgz* where *ρ* is the rock density, *g* is the gravitational acceleration and *z* is the depth. In addition, Δσ = Δ*P*_o_/P* therefore Δ*P*_o_ = ΔσP*. Therefore, the final equation is:

7 $$P_{{\text{f}}} = \sigma_{{\text{v}}} - \Delta \sigma + Ts + \Delta \sigma P^*.$$

## Geological setting and extensional quartz veins around Nobeoka Thrust

**Figure 2:**
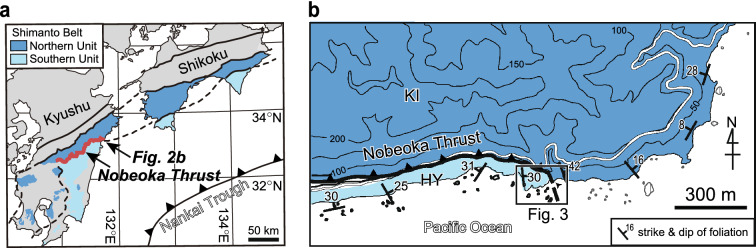
Geological conditions on the Nobeoka Thrust. (**a**) Geological setting of the Nobeoka Thrust, southwest Japan^48^. (**b**) Geologic map of the studied area^11^. KI: Kitagawa Group. HY: Hyuga Group. The maps were created using Adobe Illustrator CC software.

**Figure 3:**
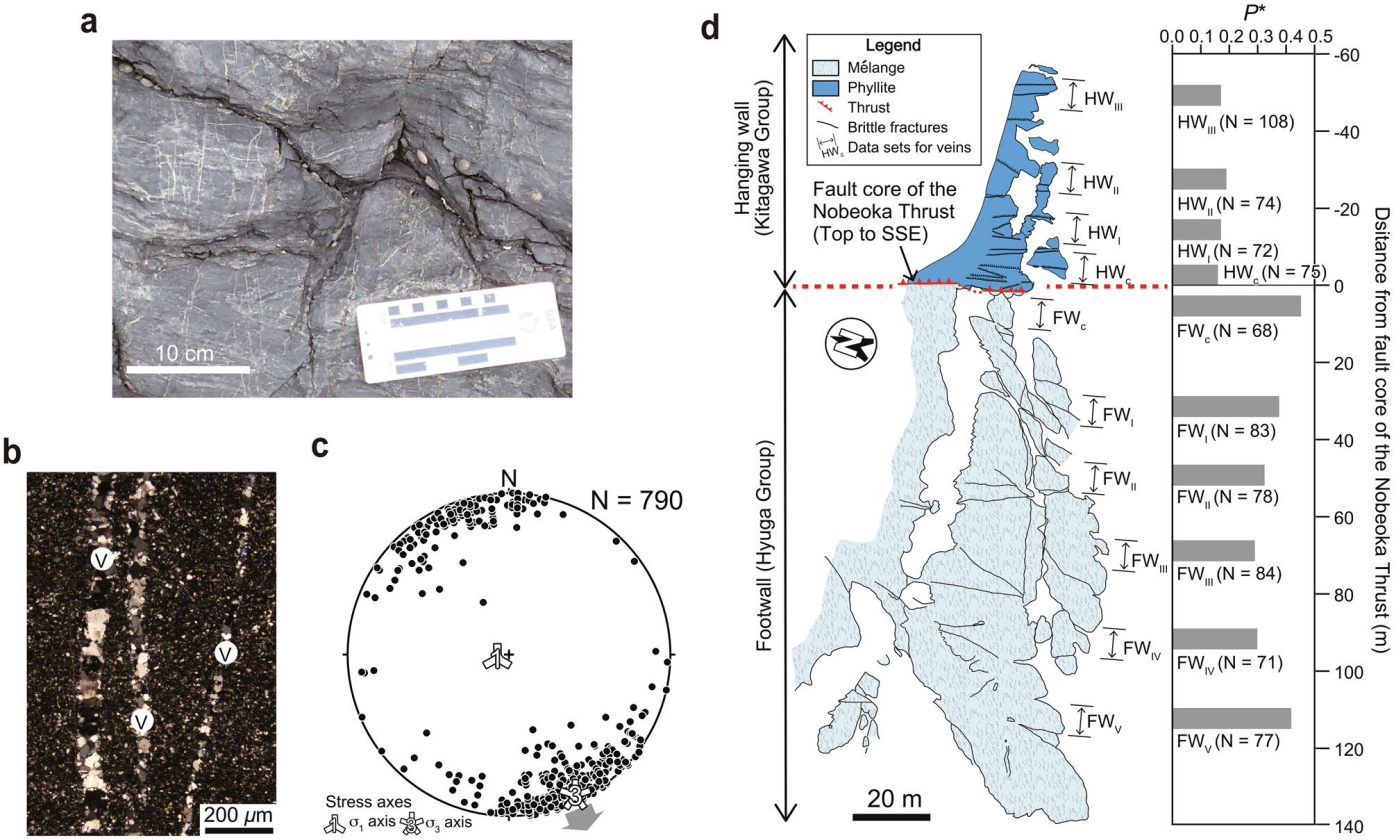
The formation of extension veins associated with thrust. (**a**) Photograph of the extension quartz vein observed around the Nobeoka Thrust. (**b**) Photograph of a thin section from the quartz veins around the Nobeoka Thrust. V: vein. (**c**) Equal-area projection showing poles to the extension veins around the Nobeoka Thrust^13^. N is number of the veins. σ_1_ and σ_3_ are maximum and minimum principal stresses, respectively, detected by using the stress tensor inversion^13^. Gray arrow indicates the slip direction of the Nobeoka Thrust (top-to-the-SSE^10^). (**d**) Geological map of the coastal region of the Nobeoka Thrust and driving pore fluid pressure ratio (P*) around the Nobeoka Thrust inferred from the stress tensor inversion^13^. N is number of the veins. The westward distance from the fault core of the Nobeoka Thrust is represented by a positive distance. The map was created using Adobe Illustrator CC software.

We observed discrete extensional veins around the Nobeoka Thrust, filled mainly with quartz and lesser calcite^13^. The Nobeoka Thrust is a major fault that bounds the northern and southern Shimanto belts of Kyushu, southwestern Japan^10^ (Fig. 2, traceable for > 800 km in the Cretaceous–Neogene accretionary complex parallel to the modern Nankai Trough^10^. The Nobeoka Thrust is considered to be a fossilized seismogenic megasplay fault due to the presence of pseudotachylite in the damage zone of its hanging wall^30^ and its paleo-temperatures (hanging wall: ~ 320 °C and footwall: ~ 250 °C)^10^. Its total estimated displacement of ~ 8.6–14.4 km, based on a 70 °C temperature difference between the hanging wall (phyllite that include thin coherent sandstone layers of the Kitagawa Group) and footwall (shale-dominated chaotic rocks of mélange in the Hyuga Group), is comparable to the deeper part (~ 8 km) of the modern megasplay fault in the Nankai subductione zone^10,12^. Around the Nobeoka Thrust, we observed ~ 800 quartz veins that filled mode I crack (Fig. 3). Quartz crystals in vein grew on quartz grain surfaces, from vein walls towards vein center, and there is no evidence of repeated crack–seal events^18^ (Fig. 3a,b). Here, we interpret the quartz veins as pre-existing cracks that have been opened. *W*/*L* has been determined for quartz veins that meet the following criteria^19^: (i) mode I crack veins with no appreciable shear displacement; and (ii) veins that do not intersect other cracks, veins or rock discontinuities (unrestricted veins). *W* and *L* of the quartz veins around the Nobeoka Thrust were estimated from the thin sections and field surveys, respectively; both measurements are log-normally distributed, with *W* = 10–400 μm (geometric mean 52 μm) and *L* = 1–50 cm (geometric mean 7.4 cm), with a few veins being longer than 50 cm^18^. Overall, *W*/*L* ratios of all veins vary between 0.00002 and 0.04 with an average of 0.0007.

The stress tensor inversion reveals that the extensional quartz veins formed under a normal faulting type stress regime, with the orientation of the minimum principal stress (σ_3_) axis being sub-horizontal and trending roughly NNW–SSE in both the hanging wall and footwall^13^. The reverse faulting type stress regime in pre-seismic period, combined with the σ_3_ axis sub-parallel to the slip direction of the Nobeoka Thrust (top to the SSE)^13,31^ (Fig. 3c), indicates that the normal faulting type stress regime at the time of crack opening was a secondary stress state generated by slip of the Nobeoka Thrust^13^. Under this stress state, the increasing of pore space by the extension cracking around the Nobeoka Thrust contributes to the pore fluid pressure reduction. And, the stress tensor inversion estimated P* = 0.16–0.19 and 0.29–0.46 in the hanging wall and footwall, respectively^13^ (Fig. 3d).

## Pore fluid overpressure in postfailure period around Nobeoka Thrust

**Figure 4:**
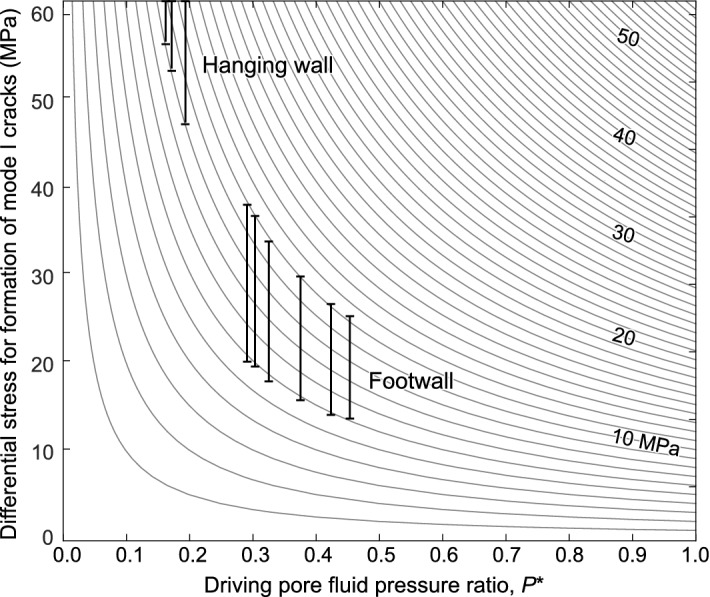
Pore fluid overpressure (Δ*P*_o_) for formation of mode I cracks around the megasplay fault, Nobeoka Thrust. Pore fluid overpressure (Δ*P*_o_) is represented by the gray contours. Black line bars represent the range of the fluid pressure ratio (P*) and differential stress for the hanging wall and footwall of the Nobeoka Thrust, respectively.

Δ*P*_o_ at the time of vein formation can be estimated by using the average values of the vein *W*/*L* ratio. *E* varies from 25–51 and 15–30 GPa for the rocks of the hanging wall and footwall respectively^32^, whereas *ν* and *Ts* of the rocks of the hanging wall and footwall are not known directly. We therefore adopt values derived from the elastic parameters of lithologies that best approximate the mechanical behavior of this unit (i.e., shale, sandstone and phyllite). The following elastic parameters were derived from the literature: ν = 0.25 and *Ts* = 15 Ma (hanging-wall) and 10 MPa (footwall)^33–36^. The resulting average Δ*P*_o_ values range between ~ 9 and ~ 19 MPa and between ~ 6 and ~ 11 MPa for the hanging wall and footwall veins, respectively (Fig. 4). The average Δσ values range between ~ 47 and ~ 119 MPa and between ~ 15 and ~ 40 MPa for the hanging wall and the footwall veins, respectively (Fig. 4). Here, because the extensional veins were formed as mode I cracks under low differential stress of Δσ < 4*Ts* ^21^, Δ*P*_o_ and Δσ for the hanging wall can be ~ 9–~ 12 MPa and ~ 31–~ 60 MPa, respectively. Hence, the maximum case of Δ*P*_o_ for the hanging wall and footwall are 12 MPa and 11 MPa, respectively. Taking into account that *P*_f_ = σ_v_ − ∆σ + *Ts* + ∆σP* and assuming that *ρ* = 2,700 kg/m^3^ (the rocks of the hanging wall and footwall^37^) at 8 km depth (*z* = 8 km), the *P*_f_ derived from our vein data range between ~ 189 and ~ 196 MPa and between ~ 200 and ~ 211 MPa for the hanging wall and the footwall veins, respectively. Hence, the maximum case of *P*_f_ for the hanging wall and footwall are 196 MPa and 211 MPa, respectively.

## Temporal change of pore fluid pressure around Megasplay fault

**Figure 5:**
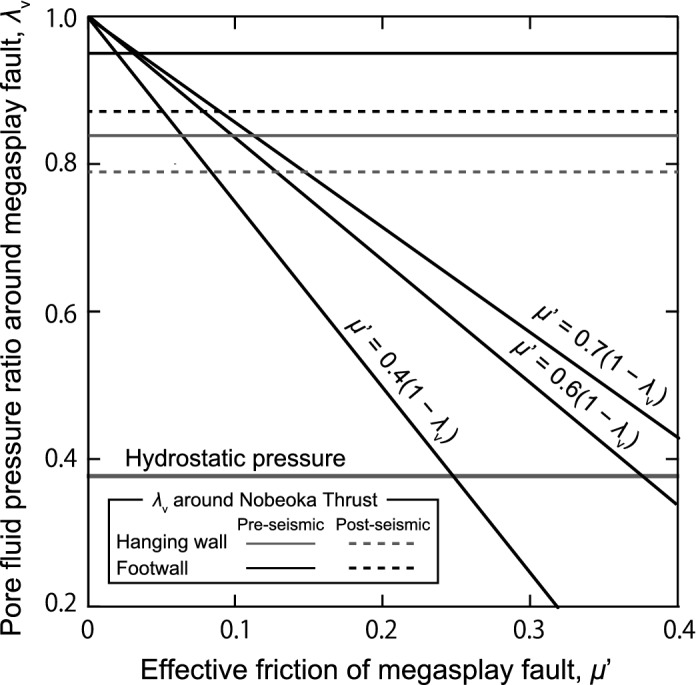
A diagram for effective frictional coefficient along the megasplay fault. Pore fluid pressure ratio in the hanging wall and footwall of the Nobeoka Thrust is represented by horizontal gray and black lines, respectively. Hydrostatic pressure around the megasplay is indicated by a horizontal gray thick line. Thick lines are the effective frictional coefficient *μ′* (*μ′* = *μ*_0_(1 − *λ*_v_)) for *μ*_0_ = 0.4, 0.6 and 0.7.

We show that the pore fluid pressure at the mode I crack formation is close to the lithostatic pressure around the Nobeoka Thrust. In the maximum case of footwall of the Nobeoka Thrust at 8 km depth, the pore fluid pressure ratio (*λ*_v_ = *P*_f_/σ_v_) for the formation of the mode I crack exceeds 0.95. To compare with the pore fluid pressure inferred from the seismic velocity structures, this ratio can be converted to the normalized fluid pressure ratio, λ*, which is calculated as follows:

8 $$\lambda^* = \left( {P_{{\text{f}}} - P_{{\text{h}}} } \right)/\left( {{\text{P}}_{{\text{c}}} - P_{{\text{h}}} } \right)$$

where *P*_h_ is the hydrostatic pressure and *P*_c_ is the lithostatic pressure^25,38^. The calculate λ* is 0.92. When the high-pressure fluid flows into the mode I cracks, the overpressure in the total pore fluid pressure is reduced. In the maximum case of the Nobeoka Thrust (8 km depth), ~ 7% and ~ 8% of the total pore fluid pressure, ~ 12 MPa and ~ 11 MPa, are reduced by the formation of the mode I cracks in the hanging wall and footwall of the Nobeoka Thrust, respectively. When the pore fluid pressure falls below the sum of σ_3_ and *Ts*, the cracks are closed and the reduction of pore fluid pressure is stopped^26^. Here, for the Eq. (8), we use the maximum cases of the pore fluid pressure for the hanging wall and footwall to estimate the temporal changes of the normalized pore fluid pressure ratio λ* and the pore fluid pressure ratio *λ*_v_. During the formation of the mode I crack in the post-seismic period, the normalized pore fluid pressure ratio λ* (the pore fluid pressure ratio *λ*_v_) changes from 0.75 to 0.66 (from 0.84 to 0.79) and from 0.92 to 0.80 (from 0.95 to 0.87) in the maximum cases of hanging-wall and footwall of the Nobeoka Thrust, respectively (Fig. 5). The results indicate that there is small change in the pore fluid pressure around the Nobeoka Thrust during the seismic cycle.

We compare our results with the pore fluid pressure around the currently-active megasplay fault in the Nankai Trough. The fluid pressure ratio *λ*_v_ along the seismogenic zone in the Nankai Trough is as high as 0.95–0.98, which can be converted to normalized fluid pressure ratio of λ* = 0.92–0.96^3^. The maximum case of our results during the pre-seismic period support similar *λ*_v_ in the Nankai Trough^3^. The value of normalized pore fluid pressure ratio λ* around the megasplay fault in the Nankai Trough is around 0.8, inferred from the seismic velocity structures^39^. The value of λ* after the large earthquakes that we estimated in this study is consistent with the value revealed by the seismic velocity structures^39^. Hence, our results indicate the temporal variation of the pore fluid pressure along the megasplay fault at a depth of ~ 8 km beneath the sea bottom surface in the seismogenic zone. In the present Nankai Trough, ~ 70 years have passed since the last large earthquake, the 1944 Tonankai Earthquake (M = 7.9). Our results indicate that the present pore fluid pressure around the megasplay fault in the Nankai Trough has not recovered to the value of the pre-seismic period.

Cracks opened in the post-seismic period are closed by the precipitation of silica in the pore fluid^6,18,40^. In the subduction zone, the fluid can be supplied to the upper plate by the dehydration from subducting sediments and altered oceanic crust^41–44^. Saishu et al.^18^ proposed a novel model of quartz vein formation associated with fluid advection from the host rocks and silica precipitation in a crack. In the extension quartz veins around the Nobeoka Thrust, the fluid pressure drops of 10–25 MPa from the lithostatic pressure facilitates the formation of the extension quartz veins under a period of 50–100 years^18^. When the cracks are closed by the silica, the porosity of the host rock decreases. The occlusion of the porosity promotes the build-up of the pore fluid pressure. Hence, the sealing time of silica in the cracks around the Nobeoka Thrust^18^ suggests that the onset of increasing of pore fluid pressure around the megasplay fault is delayed from the start of tectonic stress accumulation.

## Limited increase of fault strength after formation of mode I cracks

The fluid loss by the formation of mode I cracks increases the fault strength and creates drainage asperities along plate interface^6^. Approximating the frictional strength of the megasplay fault to *τ*_f_ = *μ*_0_σ_v_′ = *μ*_0_*ρgz*(1 − *λ*_v_) and adopting a frictional coefficient (*μ*_0_) of 0.6 (Byerlee's friction law^23^) and a density (*ρ*) of 2,700 kg/m^3^ (the rocks of the hanging wall and footwall^34^), any increases in frictional strength (Δ*τ*_f_), can be related to decreases in *P*_f_. When the pore fluid pressure decreases by ~ 11–19 MPa due to the extension crack formation, frictional strength can increase by ~ 7–12 MPa. The order of pore fluid pressure change (Δ*P*_f_) and increasing Δ*τ*_f_ is less than the stress drop in large trench type earthquakes (20–40 MPa^45^). The temporal change in fault frictional strength is at the low end of the estimates of Sibson^6^ for 10 km depth. Here, the effective frictional coefficient (*μ*′) of the megasplay fault is estimated by *μ*′ = *μ*_0_(1 − *λ*_v_). Previous studies showed that the frictional coefficient (*μ*_0_) of illite-rich shales ranges from ~ 0.4 to ~ 0.7^46^ at room temperature. In this study, we employed a frictional coefficient (*μ*_0_) of 0.4–0.7 along the Nobeoka Thrust. In the case of the Nobeoka Thrust, the effective frictional coefficient (*μ*′) before the fluid-loss by the formation of mode I cracks is smaller than 0.12 (Fig. 5). After the fluid-loss, the effective frictional coefficient (*μ*′) increases up to 0.15 (Fig. 5). The results indicate that the change amount of the pore fluid pressure by formation of mode I cracks is too small to substantially change the fault strength of the megasplay fault (Fig. 5). Hence, we suggest that the reduction of pore fluid pressure due to the mode I crack formation at post-seismic period plays limited contribution to the increasing of the effective frictional coefficient along the megasplay fault. In the hanging wall, the relative expansion following a megathrust earthquake may reduce the fluid pressure. Flow from a highly-pressured fault interface into these zones would seem to have the potential to reduce fluid pressure on the fault interface. Future work will focus on relationships between the upwelling drainage by the extension crack formation and the expansion.

In summary, our model offers an explanation for how much pore fluid pressure decreases by the extensional crack formation during post-seismic period. It implies that the pathways of upwelling drainage by the extension crack formation are the limited deformation process of the strength-cycling (Figs. 4 and 5). The earthquakes may nucleate at local heterogeneities where the pore fluid pressure is close to the lithostaic pressure^47^. The long-term monitoring of such transient pore fluid pressure variation in the order of 10^0^–10^1^ MPa between co-seismic and inter-seismic periods around fault zones is an important goal of earthquake hazard mitigation. Our results are part of a complex seismic cycle involving change in stresses orientation. Other possible component of the stress-fluid cycle is, for instance, the poro-elastic responses due to change in mean stress and the change in the permeability of the system^6^.

## Supplementary information

Supplementary file1 (DOCX 141 kb)

## Data Availability

The datasets generated during the current study are available from the corresponding author upon request.
