# Supplementary material for: Localized fluid discharge by tensile cracking during the post-seismic period in subduction zones
Source: Sci Rep. 2020 Aug 3;10:12281. doi: 10.1038/s41598-020-68418-z (PMC7398914; doi:10.1038/s41598-020-68418-z)
Supplement: Supplementary file 1 — Supplementary file1 (DOCX 141 kb) [file 41598_2020_68418_MOESM1_ESM.docx]

Localized fluid discharge by tensile cracking during the post-seismic period in subduction zones

Makoto Otsubo^1,^ *, Jeanne L. Hardebeck^2^, Ayumu Miyakawa^1^, Asuka Yamaguchi^3^, Gaku Kimura^4^

^1^Geological Survey of Japan, AIST, Tsukuba 3058567, Japan

^2^U.S. Geological Survey, Menlo Park 94025, USA

^3^The University of Tokyo, Kashiwa 2778564, Japan

^4^Tokyo University of Marine Science and Technology, Tokyo 1088477, Japan

*correspondence and requests for materials should be addressed to M.O. (e-mail: otsubo-m@aist.go.jp)


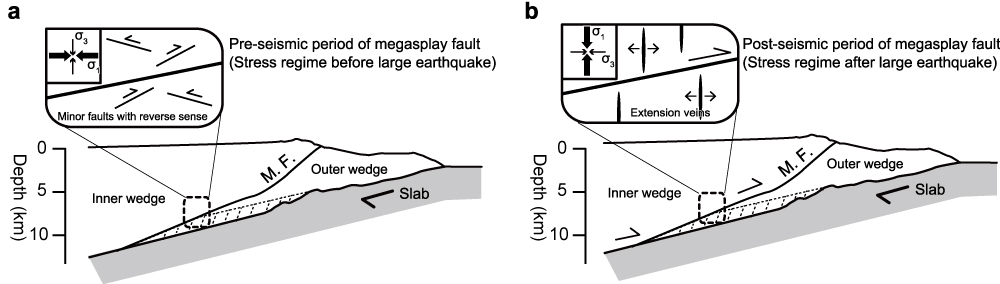


**Figure S1** | **A schematic model of spatial changes in stress regimes along the subduction zone.** (a) Pre-seismic stress regime before^1^ and (b) Post-seismic stress regime after a large earthquake due to a drop in shear stress along the megasplay fault^2^.

References

1. Kawasaki, R. *et al*. Temporal stress variations along a seismogenic megasplay fault in the subduction zone: an example from the Nobeoka Thrust, southwestern Japan. *Island Arc* **26**, e12193 (2017).
2. Otsubo, M. *et al*. Variation of stress and pore fluid pressure using vein orientation along seismogenic megasplay fault - example of Nobeoka Thrust, southwestern Japan. *Island Arc* **25**, 421–432 (2016).
